# Neutrophil-to-lymphocyte ratio: A potential new peripheral biomarker of suicidal behavior

**DOI:** 10.1192/j.eurpsy.2019.20

**Published:** 2020-02-17

**Authors:** Ángela Velasco, Julia Rodríguez-Revuelta, Emilie Olié, Iciar Abad, Abel Fernández-Peláez, Aurélie Cazals, Sébastien Guillaume, Lorena de la Fuente-Tomás, Luis Jiménez-Treviño, Laura Gutiérrez, Paz García-Portilla, Julio Bobes, Philippe Courtet, Pilar A. Sáiz

**Affiliations:** 1 Department of Psychiatry, University of Oviedo, Oviedo, Spain; 2 Biomedical Research Networking Centre in Mental Health (CIBERSAM), Oviedo, Spain; 3 Instituto de Investigación Sanitaria del Principado de Asturias (ISPA), Oviedo, Spain; 4 Mental Health Services of Principado de Asturias (SESPA), Oviedo, Spain; 5 Department of Emergency Psychiatry and Post-Acute Care, Lapeyronie Hospital, CHU Montpellier—Inserm U1061, University of Montpellier, Montpellier, France; 6 Fondation FondaMental, Créteil, France; 7 Department of Medicine, University of Oviedo, Oviedo, Spain

**Keywords:** Major depressive disorder, neutrophil-to-lymphocyte ratio, suicide vulnerability, trait marker

## Abstract

**Background.:**

Neutrophil-to-lymphocyte ratio (NLR), monocyte-to-lymphocyte ratio (MLR), and platelet-to-lymphocyte ratio (PLR) have emerged as important peripheral inflammatory biomarkers. Recent data suggest a possible role of the immune system in the pathophysiology of suicidal behavior (SB). The aim of this study is to evaluate the association among NLR, MLR, and PLR and SB in patients with major depressive disorder (MDD), and to test its validity as a biomarker for suicidality.

**Methods.:**

We evaluated 538 patients with MDD (mean age [standard deviation] = 43.87 [14.36] years; females: 68.8%). A logistic regression model was estimated to determine the independent factors associated with suicide risk in patients with and without a history of suicide attempt (SA).

**Results.:**

Three hundred ninety-three patients (74.7%) had a personal history of SA. Patients with a previous SA were more frequently female (71.9% vs. 59.6%; *p* = 0.007), significantly younger (41.20 vs. 51.77 years; *p* < 0.001), had lower depression severity at enrolment (15.58 vs. 18.42; *p* < 0.000), and significantly higher mean NLR and PLR ratios (2.27 vs. 1.68, *p* = 0.001; 127.90 vs. 109.97, *p* = 0.007, respectively). In the final logistic regression model, after controlling for age, sex, and depression severity, NLR was significantly associated with SB (*β* = 0.489, *p* = 0.000; odds ratio [95% confidence intervals] = 1.631 [1.266–2.102]). We propose a cut-off value of NLR = 1.30 (sensitivity = 75% and specificity = 35%).

**Conclusions.:**

Our data suggest that NLR may be a valuable, reproducible, easily accessible, and cost-effective strategy to determine suicide risk in MDD.

## Introduction

Suicide is a pressing public health problem. On an average, 1 million people commit suicide each year worldwide, and suicide is the second leading cause of death in the 15–29-year age group. However, this rate may be even higher since, for each suicide death, it is estimated that there are at least 20 suicide attempts (SAs) [1].

Clinical factors (especially depression and alcoholism), previous SAs, and stressful life events are generally assumed to be the best predictors of suicidal behavior (SB) [2]. Thus, clinical scales are a central component of routine clinical assessment, despite limited evidence of their effectiveness for predicting suicide risk [3]. The short-term variability of suicide ideation may be related to this failure, and some authors have suggested the need for real-time evaluation of interpersonal and psychological variables [4].

A wide range of possible biological risk factors for future SB have also been proposed. However, we are still far from having good biomarkers for SB [5], and prediction of future suicidality is currently a long-range goal. This lack of progress highlights the need for new research strategies.

Neuroinflammatory processes are an essential pathophysiologic mechanism of chronic diseases including severe mental disorders [6,7]. There is now evidence that major depressive disorder (MDD) is characterized by activation of the immune-inflammatory response system, as indicated by increased levels of pro-inflammatory cytokines including interleukin (IL)-6 and IL-10 [8]. A comprehensive model focusing on immune system influence on the pathophysiology of SB has been proposed [9]. In this model, childhood abuse, sleep disturbance, infections, and other stressors induce a chronic inflammatory state, causing dysregulation of the hypothalamic–pituitary–adrenal axis. The subsequent cortisol and indolamine 2,3-dioxygenase activation affects serotonin metabolism and increases N-methyl-d-aspartate (NMDA) agonist levels [10]. Thus, inflammatory markers could potentially be used to predict and monitor suicide risk in patients with mental disorder [11,12].

In recent years, neutrophil-to-lymphocyte ratio (NLR), monocyte-to-lymphocyte ratio (MLR), and platelet-to-lymphocyte ratio (PLR) have been found to be attractive, convenient, and cost-effective blood indicators of inflammatory status. These ratios have been used as inflammation/immune-based prognostic scores in different systemic diseases such as cancer, coronary heart disease, and pancreatitis [13].

Recently, a few studies have found NLR and PLR to be elevated in MDD [14,15] as well as bipolar disorder [16]. Furthermore, meta-analytic evidence has also shown increased NLR in patients with MDD as compared with healthy controls [17]. Two recent studies even suggest that NLR may be a marker for suicide vulnerability in patients with bipolar disorder or MDD [18,19]. NLR was substantially higher in suicidal depressed patients compared with nonsuicidal depressed patients and healthy controls. One logistic regression model included NLR and previous attempts as predictive variables for suicide status [19].

Our objective is to investigate the association among NLR, MLR, and PLR and history of SAs in patients with MDD, controlling for different potential confounding variables, in order to help determine the possible role of these ratios in predicting SB.

## Methods

### Participants

A sample of 538 Caucasian patients aged ≥18 years was recruited in the Mental Health Services of Oviedo (Asturias, Spain; *n* = 148) and in the Department of Emergency Psychiatry and Post-Acute Care at the University Hospital of Montpellier (France; *n* = 390) from September 2015 to June 2017.

All patients had a diagnosis of MDD according to Diagnostic and Statistical Manual of Mental Disorders, Fifth Edition (DSM-5) criteria. SA was also defined as a “self-initiated sequence of behaviors by an individual who, at the time of initiation, expected that the set of actions would lead to his or her own death” [20]. All patients were undergoing pharmacological antidepressant treatment at the time of the study. The exclusion criteria were comorbid psychiatric diagnoses, acute infections, active or chronic inflammatory, or autoimmune diseases, smoking ≥20 cigarettes/day, obesity (BMI > 30 kg/m^2^), current treatment with anti-inflammatory or immunosuppressive medications, acute coronary syndromes, history of chronic renal, hepatic, or cerebrovascular disease, and significant abnormalities in laboratory test results (anemia, leukocytosis, leukopenia, or thrombocytosis).

Written informed consent was obtained from all patients included in the study. The study was conducted in compliance with applicable legislation in each country and the provisions of the World Medical Association Declaration of Helsinki, and it received institutional approval [21].

### Assessments

All participants were assessed by well-trained interviewers using an “ad hoc” protocol for sociodemographic and clinical data (sex, age, pharmacological antidepressant treatment [yes/no], number of SA, and age at first attempt), as well as the Hamilton Depression Rating Scale (HDRS). Hamilton total score was evaluated as follows: 8–16: mild depression; 17–23: moderate depression; and ≥24: severe depression [22].

All the researchers involved in the clinical assessments at both sites received training in order to achieve the same standards.

For the exploratory analysis, patients with a previous SA were divided into two different groups: those with a very recent SA (≤7 days) and those with a less recent lifetime history of SA.

Fasting peripheral blood samples were collected from the cephalic vein between 8:00 and 9:00 a.m. in EDTA tubes. Complete blood counts were performed using a Sysmex XN-10/XN-20 Hematology Analyzer (Norderstedt, Germany). Complete blood counts included total white blood cells, neutrophils, lymphocytes, monocytes, and platelets. NLR, MLR, and PLR were calculated.

### Statistical analyses

The data were analyzed using SPSS 15.0 (SPSS, Inc., Chicago, IL). Data are presented as mean (standard deviation [SD]) for numeric variables and as frequencies and percentages for categorical variables.

A Kolmogorov–Smirnov normality test was used to determine if variables were normally distributed. A chi-square (*χ* ^2^) test was used to compare categorical variables and frequencies. Continuous variables were expressed as mean (SD). Normally and abnormally distributed variables were analyzed. A Student’s *t*-test and one-way analysis of variance with post hoc Duncan test was used to compare normally distributed variables between two and three groups, respectively. However, a Mann–Whitney *U* test or Kruskal–Wallis test was performed to analyze any abnormally distributed variables. Bivariate correlations were performed to determine differences according to previous SA based on sociodemographic and clinical data. A logistic regression model (forward stepwise selection) was estimated to determine the independent factors associated with suicide risk in patients with and without a history of SA. A multinomial logistic regression model (main effects model) was used to determine factors linked to suicidality in patients with MDD (“never attempted” was used as the category of reference). A receiver operating characteristic (ROC) curve analysis was performed to determine the cut-off level for biomarkers to detect the SA. The level of statistical significance was set at *α* = 0.05 (two-sided).

## Results

The final sample included 538 patients with a DSM-5 MDD diagnosis (mean age [SD] = 43.87 [14.36] years; females: 370 [68.8%]). All patients included in the study were experiencing an active episode of depression with a score of 7 or more on the HDRS scale. Severity of depression was moderate/severe in 44.8% of patients according to the HDRS score. An average NLR ratio (SD) of 2.12 (1.98) was observed in the total sample, whereas the mean MLR and PLR ratios (SD) were 0.27 (0.14) and 123.37 (55.68), respectively (Table 1).

**Table 1.** Sociodemographic and clinical characteristics of the study group

	Total sample	Recent SA (≤7 days)	Lifetime SA (≥8 days)	No history of SA	*χ* ^2^/ANOVA^a^/Student’s *t*-test^b^/Kruskal–Wallis test^b^	*p*
	*n* = 538	*n* = 126	*n* = 276	*n* = 136		
Sociodemographic data						
Sex (*n* [%)]						
Males	168 (31.2%)	36 (28.6%)	77 (27.9%)	55 (40.4%)	7.214	0.027
Females	370 (68.8%)	90 (71.4%)	199 (72.1%)	81 (59.6%)		
Age (mean [SD])	43.87 (14.36)	39.99 (14.64)^1^	41.75 (13.37)^2^	51.77 (13.10)^1,2^	31.397^a^	0.000
Clinical characteristics						
Severity of depression (mean [SD])	16.30 (5.13)	14.33 (4.07)^1,2^	16.15 (5.14)^1,3^	18.42 (5.21)^2,3^	22.803^a^	0.000
HDRS 8–16 (mild) (*n* [%])	297 (55.2%)	96 (76.2%)	154 (55.8%)	47 (34.6%)	52.996	0.000
HDRS 17–23 (moderate) (*n* [%])	188 (34.9%)	26 (20.6%)	100 (36.2%)	62 (45.6%)		
HDRS ≥24 (severe) (*n* [%])	53 (9.9%)	4 (3.2%)	22 (8.0%)	27 (19.9%)		
Number of SA (mean [SD])	–	2.79 (3.89)	2.53 (2.72)	–	0.769^b^	0.443
Age of first SA (mean [SD])	–	32.33 (14.93)	32.75 (13.87)	–	−0.273^b^	0.785
NLR (mean [SD])	2.12 (1.98)	2.37 (2.36)^1^	2.22 (2.17)^2^	1.68 (0.80)^1,2^	10.630^c^	0.005
MLR (mean [SD])	0.27 (0.14)	0.28 (0.16)	0.27 (0.15)	0.25 (0.10)	1.114^c^	0.573
PLR (mean [SD])	123.37 (55.68)	128.20 (61.65)	127.76 (58.91)^1^	109.97 (38.75)^1^	7.487^c^	0.024
Leukocytes (mean [SD])	7.15 (4.05)	7.12 (2.65)	7.24 (5.17)	6.97 (2.03)	0.061^c^	0.970
Neutrophils (mean [SD])	4.01 (2.14)	4.22 (2.79)	4.05 (2.10)	3.75 (1.39)	1.024^c^	0.599
Monocytes (mean [SD])	0.53 (0.19)	0.52 (0.20)	0.51 (0.19)^1^	0.56 (0.18)^1^	8.993^c^	0.011
Lymphocytes (mean [SD])	2.22 (0.91)	2.15 (1.10)^1^	2.16 (0.87)^2^	2.42 (0.77)^1,2^	14.482^c^	0.001
Platelets (mean [SD])	243.02 (67.19)	236.34 (60.64)	245.36 (74.31)	244.49 (57.00)	1.584^c^	0.453

Abbreviations: HDRS, Hamilton Depression Rating Scale; MLR, monocyte-to-lymphocyte ratio; NLR, neutrophil-to-lymphocyte ratio; PLR, platelet-to-lymphocyte ratio; SA, suicide attempt; SD, standard deviation; *χ* ^2^, chi-square test.

Note: Superscript numerals represent statistically different groups after post hoc Duncan test.

a One-way analysis of variance (ANOVA).

b Student’s *t*-test.

c Kruskal–Wallis test.

There were 402 patients (74.7%) with a personal history of SA (mean age [SD] = 41.20 [13.78] years; females: 289 [71.9%]). Mean age (SD) at first SA was 32.62 (14.20) years and mean number (SD) of SA was 2.62 (3.13). Patients with no history of SA were more frequently males (40.4% vs. 28.6 and 27.9%; *χ* ^2^ [df] = 7.214 [2], *p* = 0.027), were significantly older (51.77 [13.10] vs. 39.99 [14.64] and 41.75 [13.37] years; *F* = 31.397, *p* < 0.001), had higher depression severity (HDRS score: 18.42 [5.21] vs. 14.33 [4.07] and 16.15 [5.14]; *χ* ^2^ [df] = 32.168 [2], *p* < 0.001), and had significantly lower mean NLR and PLR ratios (1.68 [0.80] vs. 2.37 [2.36] and 2.22 [2.17], *H* = 10.630, *p* = 0.005; 109.97 [38.75] vs. 128.20 [61.65] and 127.76 [58.91], *H* = 7.487, *p* = 0.024, respectively; Table 1).

No association was found between NLR or PLR and age (*r* = 0.002, *p* = 0.957 and *r* = 0.046, *p* = 0.290, respectively), number of SA (*r* = −0.062, *p* = 0.222 and *r* = 0.041, *p* = 0.422), or age at first SA (*r* = 0.060, *p* = 0.242 and *r* = −0.004, *p* = 0.942). No association was found between NRL and depression severity (*r* = −0.015, *p* = 0.730), whereas PRL was significantly associated with depression severity (*r* = −0.113, *p* = 0.009). However, both ratios were associated with sex with NLR higher in men (2.37 vs. 1.99; *U* = 26,730, *p* = 0.009) and PLR higher in women (127.25 vs. 114.80; *U* = 35,248.50, *p* = 0.013). Conversely, MLR was associated with age (*r* = 0.096, *p* = 0.026) and age at first SA (*r* = 0.119, *p* = 0.020), whereas no association was found between MLR and depression severity (*r* = −0.045, *p* = 0.298), number of SA (*r* = −0.048, *p* = 0.344), or sex (*U* = 28,107, *p* = 0.094). A significant correlation was found between NLR and PLR (*r* = 0.462, *p* < 0.001), NLR and MLR (*r* = 0.518, *p* < 0.001), and MLR and PLR (*r* = 0.607, *p* < 0.001).

Since no statistically significant differences were found between the two SA subgroups, we used SA (yes/no) as a dependent variable for the stepwise logistic regression model. The model was run to assess the potential predictive value of NLR and PLR on SA. After controlling for age, sex, and depression severity, only NLR (*β* = 0.489, *p* < 0.001; odds ratio [OR] [95% confidence intervals (CI)] = 1.631 [1.266–2.102]) was included in the final model.

The multinomial regression analysis shows no differences in NLR or PLR between recent and past attempters, while patients with MDD who never attempted suicide exhibited significantly lower NRL compared with recent attempters (*β* = 0.471, *p* = 0.003; OR [95% CI] = 1.602 [1.177–2.179]) and past attempters (*β* = 0.423, *p* = 0.006; OR [95% CI] = 1.526 [1.131–2.059]; Table 2).

**Table 2.** Variables associated with never attempted versus recent attempt or past attempt

	*β*	SE	Wald	df	*p*	OR	95% CI
Recent attempt							
Intersection	4.943	0.786	39.540	1	0.000		
PLR	0.001	0.003	0.107	1	0.743	1.001	0.995–1.008
NLR	0.471	0.157	8.999	1	0.003	1.602	1.177–2.179
HDRS	−0.165	0.029	32.703	1	0.000	0.847	0.801–0.897
Age	−0.068	0.011	41.394	1	0.000	0.934	0.915–0.954
Sex (male)	−0.638	0.302	0.035	1	0.035	0.528	0.292–0.955
Past attempt							
Intersection	4.034	0.676	35.583	1	0.000		
PLR	0.002	0.003	0.522	1	0.470	1.002	0.996–1.008
NLR	0.423	0.153	7.655	1	0.006	1.526	1.131–2.059
HDRS	−0.081	0.023	12.861	1	0.000	0.922	0.882–0.964
Age	−0.059	0.009	41.833	1	0.000	0.943	0.926–0.960
Sex (male)	−0.592	−0.249	5.641	1	0.018	0.553	0.340–0.902

Abbreviations: CI, confidence interval; df, degrees of freedom; HDRS, Hamilton Depression Rating Scale; NLR, neutrophil-to-lymphocyte ratio; OR, odds ratio; PLR, platelet-to-lymphocyte ratio; SE, standard error.

**Figure 1.**
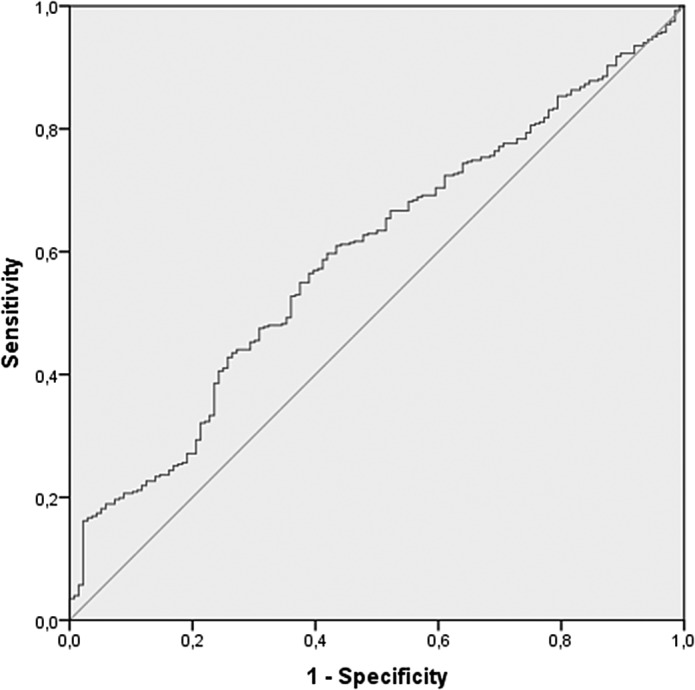
Receiver operating characteristic (ROC) curve of neutrophil-to-lymphocyte ratio for prediction of suicide attempt.

According to the ROC curve analysis (Figure 1), the optimal cut-off value for NLR in predicting SA was 1.59 (area under the curve = 0.593 [95% CI = 0.540–0.646], sensitivity = 60%, and specificity = 58%). However, in order to reduce the large number of false negatives, we suggest a cut-off of 1.30 (sensitivity = 75%, specificity = 35%, positive predictive value = 77%, and negative predictive value = 32%).

## Discussion

### Inflammation and suicidality

In the present study, we suggest a role of inflammation in SB that may be detected by white blood cell count. NLR was higher in suicidal versus nonsuicidal depressive patients and may represent a marker of SA in patients with MDD. Interestingly, NLR was associated with SA after controlling for age, sex, and depression severity.

A relationship between different chronic inflammatory states and depression or SB has already been suggested in previous research. First, autoimmune disorders such as multiple sclerosis and systemic lupus erythematosus have been associated with a twofold increase in risk of death by suicide [23] and higher prevalence of suicidal ideation [24]. Second, the prevalence of suicidal ideation and SA is higher in HIV patients and chronic hepatitis C patients [25], and *Toxoplasma gondii* infection has been associated with SB [26]. It is worth noting that infections activate neuroinflammatory mechanisms associated with the pathophysiology of SB. Third, acute-phase inflammatory markers such as C-reactive protein (CRP) have been associated with SB [27,28]. Fourth, the therapeutic benefit of ketamine for reducing suicidal ideation may be related, at least in part, to its NMDA antagonist effect [29]. It is also noteworthy that increased NMDA agonist levels seem to be affected by inflammatory processes [10].

Interoceptive signaling of inflammation also plays a role in human depression. Cumulative meta-analyses now provide convincing evidence for elevated peripheral inflammatory markers, particularly IL-6 and CRP, in a subset of depressed patients [30]. Interoceptive pathways link these central and peripheral immune changes and make a powerful contribution to maladaptive stress responses such as SB.

Altogether, these studies support the hypothesis that inflammation may be a critical factor in the etiology of mood disorders and suicide risk. Thus, immunomodulatory therapies may be effective treatment options. To develop individualized therapeutic strategies, some authors point out the need for identifying subgroups of patients with psychiatric disorders and signs of immune dysregulation [31], hence the need for inflammation markers in SB.

### NLR as a biological marker of SB

Our results confirm previous findings by Ekinci and Ekinci also in a sample of MDD patients [19]. These authors suggest that NLR may be a trait marker for suicide vulnerability in MDD.

Previous studies had already shown that the NLR is significantly higher in patients with MDD [14] and bipolar disorder [16] than in healthy controls. In addition, it has recently been suggested that NLR may be useful in predicting risk of SA in the subpopulation of bipolar disorder patients with a family history of SA [18] or in differentiating violent SAs and nonviolent SAs [32]. The only negative study, to the best of our knowledge, regarding an association between NLR and suicidality is the one by Gundogdu Meydaneri and Meydaneri [33]. However, the small sample size in the study may have led to a type II error.

White blood cell and subtype counts are some of the predictors of chronic inflammation. It has been suggested that stress and depression increase leukocyte and neutrophil levels, whereas they decrease lymphocytes [34]. However, it has also been suggested that NLR is a more reliable and stable peripheral-blood biomarker of inflammation than CRP levels or lipid profile for predicting suicidality [19]. Our results strengthen this association and extend it to suicidality, as depressed patients with a lifetime history of SA showed higher neutrophil and lower lymphocyte levels than depressed patients without a history of suicidality.

In contrast, our study did not show any differences in PLR index between MDD patients with or without a history of SA. PLR has previously been correlated with severity of MDD. More specifically, patients who had MDD with psychotic features had higher PLR scores than other MDD patients [15]. The latter research, along with recent studies conducted in other areas, have reported that PLR may be better than NLR for determining severity of inflammation [35]; thus the lack of association between PLR and suicide risk found in our study was unexpected.

### NLR cut-off value

To date, this is the first study suggesting a NLR cut-off value associated with SA. Our data suggest that NLR is significantly associated with SA in MDD patients with an optimal cut-off value of 1.59 (sensitivity 60% and specificity 58%). However, after evaluating our results, we suggest that a more suitable cut-off point for evaluation of suicide risk would be 1.30. With this cut-off point, at least in our sample, there is a major increase in sensitivity (75%), although specificity falls to 35%. Nevertheless, given the consequences of the event, we wish to predict and the possibility of using NLR to screen for suicide risk in situations where time is of the essence, such as hospital emergency room or primary care settings, it is clear that good sensitivity must take priority over specificity. It should be noted that Orum et al. [32] proposed 2.22 as the optimal cut-off for NLR in order to differentiate violent from nonviolent SA (sensitivity 68.8% and specificity 60.6%).

### Strengths and limitations

Our study has some strengths and limitations. On the one hand, we collected a large cohort from two independent institutions with a sample size large enough to find a significant association between NLR and lifetime SA in patients with MDD. In addition, the fact that we used a sample from two different countries may contribute to the generalizability of our results in Caucasian populations. Patients with chronic illnesses and those treated with anti-inflammatory drugs were excluded. Finally, we controlled for age, sex, and depression severity to ensure reliable results.

One limitation of the present study is its cross-sectional design, which limits conclusions regarding causal relationships between history of SA and inflammation. Other limitations may include potential confounding factors such as tobacco use, number of previous MDD episodes, antidepressant psychiatric treatment, including dosage and time on treatment, or the lack of a healthy control group. However, we consider these partial limitations, since our study is aimed at assessing the power of NLR, PLR, and MLR to separate MDD patients based on SA, and if strong enough, to define a cut-off with prognostic value. We have observed no differences based on tobacco use or treatment among MDD patient subgroups and therefore conclude that the potential use of NLR as a prognostic biomarker should not be biased by these variables.

In conclusion, our data confirm that using NLR may be a valuable, reproducible, easily accessible, and cost-effective strategy in clinical practice for detecting suicide vulnerability in patients with MDD. To date, there is no strong biomarker associated with suicidality. In particular, it is more probable that a combination of different biomarkers could predict the risk of SBs [36]. However, prospective studies are needed in order to determine causal relationships. Studies that include other psychiatric diagnoses are also needed in order to test the specificity of our findings.

## Data Availability

There are no linked research data sets. Data will be made available on request.
